# Comment on “how the addition of selinexor/bortezomib/dexamethasone as a Category 1 recommendation may erode outcomes”

**DOI:** 10.1093/oncolo/oyag112

**Published:** 2026-04-07

**Authors:** Joshua Richter, Michael Davis, Yi Chai

**Affiliations:** Division of Hematology and Medical Oncology, Tisch Cancer Institute, Icahn School of Medicine at Mount Sinai New York, NY 10029, United States; Quillen College of Medicine, East Tennessee State University, Johnson City, TN 37604, United States; Karyopharm Therapeutics, Newton, MA 02459, United States

On July 9, 2025, a commentary in *The Oncologist* questioned the Category 1 recommendation for the triplet therapy selinexor/bortezomib/dexamethasone (SVd) in relapsed/refractory multiple myeloma (RRMM).^1^ The authors challenged the use of bortezomib/dexamethasone (Vd) as the comparator in the BOSTON trial,^2^ suggested that alternative regimens should define treatment sequencing, and argued that informative censoring may have inflated progression-free survival (PFS) results in the SVd arm. These critiques call into question both the design and the validity of the BOSTON trial, which supports SVd’s standing in the NCCN (National Comprehensive Cancer Network) Guidelines. We address these concerns by clarifying the rationale behind the trial’s comparator choice, its statistical integrity, and the ongoing clinical relevance of SVd. The evidence continues to support SVd as a Category 1 recommendation.

NCCN Guidelines^3^ are developed by over 1900 oncology experts across 63 panels from 33 member institutions. Recommendations are based on high-level evidence and consensus—Category 1 requires data from at least one randomized phase 3 trial and ≥85% panel agreement. The BOSTON trial,^2^ a multinational, randomized phase 3 study comparing SVd to Vd, demonstrated a clinically meaningful PFS improvement of more than 4 months and supported the current guideline classification.

Chahine et al. contend that Vd was an inferior comparator, given that the ENDEAVOR study (2016) showed carfilzomib/dexamethasone (Kd) superior to Vd.^4^ However, ENDEAVOR was criticized for imbalances in prior therapy exposure—54% of participants had previously received bortezomib compared with <1% for carfilzomib—raising concerns about bias.^5^ In contrast, the BOSTON trial balanced prior bortezomib exposure between arms (69% vs 70%), providing a more reliable evaluation of selinexor’s benefit in a clinically relevant population.

Selecting an appropriate comparator arm in a clinical trial is challenging—particularly in a rapidly evolving treatment landscape like RRMM. Although BOSTON began enrolling patients in 2017, the design of a phase 3 study starts well in advance, particularly when the goal is regulatory approval. Study design is reviewed and agreed upon well in advance of enrollment with health agencies such as the Food and Drug Administration (FDA) and European Medicines Agency. Even if the ENDEAVOR^4^ study was perfectly designed, there are multiple clinical reasons to choose one proteasome inhibitor over another. Carfilzomib is associated with a higher risk of grade 3-4 adverse events compared to bortezomib. In the ENDEAVOR trial, grade ≥3 adverse events occurred in 81% of carfilzomib-treated patients vs 71% with bortezomib, with higher rates of hypertension, cardiac events, and renal toxicity for carfilzomib, but lower rates of peripheral neuropathy.^4^ Meta-analyses and real-world data confirm increased cardiovascular and renal toxicity with carfilzomib, while bortezomib is more likely to cause neuropathy.^6^ Additional recent clinical trials in RRMM have been criticized for comparator arms not deemed standard of care due to the rapidly changing landscape in this disease area.^7–9^

It is important to recognize that the standard of care in multiple myeloma continues to evolve. Twice-weekly bortezomib was historically a regulatory requirement and remains embedded in many trial designs despite practice shifts. Over the past two decades, bortezomib-based induction regimens (VMp, VCd, VTd, VRd ± CD38 antibodies) have dominated frontline therapy globally. The emergence of MAIA and related data has introduced a new category of early-relapse patients—CD38-refractory or IMiD (Immunomodulatory agent)-refractory yet proteasome inhibitor–naïve.^10^ For such patients, selinexor-based combinations represent a rational and data-supported approach, aligning with evolving real-world needs and reinforcing the BOSTON trial’s enduring clinical relevance.

The commentary also asserts that higher treatment discontinuation in the SVd arm (46 vs 26 patients) might indicate informative censoring, biasing the PFS analysis. The authors cite a reverse Kaplan–Meier (KM) analysis assuming censored patients had poor outcomes. This assumption is neither standard nor supported by regulatory science. All censoring and discontinuation procedures in BOSTON adhered to ICH-GCP (International Council for Harmonisation - Good Clinical Practice) and FDA statistical guidelines. The prespecified statistical analysis plan included multiple sensitivity analyses reviewed by regulators to ensure robustness against informative censoring. None suggested that the PFS benefit for SVd was spurious or artifactual.

Treatment discontinuation is not synonymous with progression (or death). Although duration of study treatment was similar between arms, SVd led to significantly longer time to next treatment (TTNT) than Vd—median 16.13 months for SVd vs 10.84 months for Vd (HR 0.66; 95% CI: 0.50-0.86).^11^ Given that there is no survival detriment observed with SVd,^2^ if patients had progressed around the time of discontinuation, one would expect new treatment to begin shortly thereafter. The extended TTNT observed in the SVd arm supports the interpretation that many of these patients that discontinued treatment had not progressed, directly contradicting the assumption that censoring was informative.

Although ORR by censoring status is not typically reported, review of data on file indicates that among patients censored, those in the SVd arm had an ORR of 76.9% compared to 63.3% in the Vd arm. These rates were higher than the ORR (Objective Response Rate) in patients who experienced a PFS event (SVd: 66.3%; Vd: 53.2%). This indicates that SVd demonstrated superior ORR compared to Vd for both censored and uncensored patients. Since ORR is a reliable predictor of PFS in previously treated multiple myeloma, there is no compelling reason to assume that if censored patients had remained on study, the PFS results would have changed meaningfully.^12^

The validity of any reverse KM analysis depends on accurately reconstructing the original time-to-event data. The authors relied on WebPlotDigitizer and reconstructKM, which can only approximate individual patient data from published curves. Such reconstructions cannot accurately replicate the original number of events and censoring times as evidenced by the clear differences in censoring/events between the PFS curves presented in Figure 1a when compared to the original PFS curve from the BOSTON trial.^2^ It is important to note that the reverse KM method is designed for estimating follow-up time or, at most, for exploratory visualization of censoring patterns—not for recalculating PFS or for concluding that a treatment effect is eliminated.

The argument posed by Chahine et al. also ignores important clinical decision-making by practitioners due to the BOSTON trail, SVd classification, and subsequent research. Recent data suggest that selinexor may play an important “bridging” or “holding” role for patients awaiting CAR (Chimeric Antigen Receptor)-T cell therapy.^13^^,^^14^ CAR-T cell therapy is not universally available in the United States or worldwide. Despite FDA approval, access is limited by high cost, infrastructure requirements, manufacturing capacity, insurance coverage, and geographic disparities. This positioning substantially enhances the relevance of selinexor early in the treatment continuum, particularly as a pre–CAR-T option maintaining disease control and T-cell fitness prior to CAR-T. Moreover, given emerging evidence that CAR-T may function as a non-curative modality in certain patients—often leaving behind a population of T cells characterized by exhaustion secondary to both redirection and prior fludarabine/cyclophosphamide conditioning—selinexor may offer renewed therapeutic utility in the post–CAR-T setting due to its distinct mechanism of action.^15^

Characterizing certain regimens as “subpar” oversimplifies nuanced clinical decision-making. The balance of risks and benefits should always rest between clinician and patient, and regulatory approvals enable precisely this individualized approach.

Selinexor is not suitable for every patient, yet the assertion that it should be excluded at first relapse is unwarranted. Older, frailer patients who have received daratumumab/lenalidomide/dexamethasone upfront and relapse early—especially those without access to or suitability for BCMA (B cell maturation antigen)-based therapy—may benefit significantly from SVd. The heterogeneity of multiple myeloma necessitates preserving therapeutic flexibility for such individuals. While BCMA-directed therapies continue to transform the relapsed/refractory landscape, their availability remains limited in many centers due to logistical complexity and socioeconomic disparities. Ensuring equitable access to active agents like selinexor across diverse treatment settings is critical to maintaining fairness in therapeutic opportunity.

In summary, the critiques raised against the SVd regimen as a Category 1 recommendation fail to undermine the strength of the BOSTON trial’s findings or the validity of its clinical relevance. The trial was appropriately designed and executed in alignment with regulatory standards, using a comparator that was standard of care at the time. The results and interpretation were reviewed extensively and favorably by health regulatory authorities and independent expert panels, including NCCN. The comparator arm of Vd was an appropriate choice at the time of the BOSTON study, given contemporaneous available clinical information. Concerns about informative censoring do not hold under closer scrutiny; alternative sensitivity analyses either estimate different endpoints or fail to demonstrate a meaningful impact on the PFS benefit. Furthermore, additional data points such as TTNT and overall response rate reinforce the conclusion that SVd offers a clinically meaningful advantage over Vd. The evolving evidence base underscores selinexor’s enduring clinical relevance—not only as a rational and accessible option for older or frailer patients at early relapse but also as a valuable bridge and post–CAR-T therapy. Its inclusion within the treatment armamentarium ensures clinicians retain the flexibility to individualize care amid an increasingly complex and heterogeneous myeloma landscape. While continued evaluation of treatment sequencing in RRMM is important, the current evidence base supports the continued Category 1 designation of SVd in the NCCN Guidelines.

## Data Availability

No new data were generated or analysed in support of this research.
